# Enrichment of the Glycyrrhizic Acid from Licorice Roots (*Glycyrrhiza glabra* L.) by Isoelectric Focused Adsorptive Bubble Chromatography

**DOI:** 10.1155/2016/7201740

**Published:** 2016-02-01

**Authors:** Eyyüp Karaoğul, Perihan Parlar, Harun Parlar, M. Hakkı Alma

**Affiliations:** ^1^Department of Forest Industry Engineering, Faculty of Forestry, Kahramanmaraş Sutcu Imam University, Kahramanmaraş, Turkey; ^2^Faculty of Health Sciences, Istanbul Esenyurt University, Esenyurt, 34510 Istanbul, Turkey; ^3^Department of Chemical-Technical Analysis and Chemical Food Technology, Technical University of Munich, Munich, Germany

## Abstract

The main aim of this study was to enrich glycyrrhizic acid ammonium salt known as one of the main compounds of licorice roots (*Glycyrrhiza glabra *L.) by isoelectric focused adsorptive bubble separation technique with different foaming agents. In the experiments, four bubble separation parameters were used with *β*-lactoglobulin, albumin bovine, and starch (soluble) preferred as foaming agents and without additives. The enrichment of glycyrrhizic acid ammonium salt into the foam was influenced by different additive substances. The results showed that highest enrichment values were obtained from *β*-lactoglobulin as much as 368.3 times. The lowest enrichment values (5.9 times) were determined for the application without additive. After enrichment, each experiment of glycyrrhizic acid ammonium salt confirmed that these substances could be quantitatively enriched into the collection vessel with isoelectric focused adsorptive bubble separation technique. The transfer of glycyrrhizic acid ammonium salt into the foam from standard solution in the presence of additive was more efficient than aqueous licorice extract.

## 1. Introduction

*Glycyrrhiza glabra* L. (licorice) is a ligneous perennial shrub growing in Mediterranean region and Asia and widespread in Turkey, Italy, Spain, Russia, Syria, Iran, China, and Israel [1]. The licorice having multi years has blue and violet flowers [2].

Licorice is a favorable herb used in food and pharmaceutical for thousands of years in the traditional medicine system. The secondary metabolites of this plant have long been evaluated for their use in relieving respiratory ailments (such as bronchitis, allergies, cold, tuberculosis, and sore throats), their demulcent effect (soothing, coating agent), relieving stomach burn symptoms including heartburn resulting from reflux or any other cause, and treating gastritis, inflammatory disorders, liver problems, and skin diseases [3]. The licorice roots have antiulcer, expectorant, diuretic, laxative, sedative [4], antipyretic [5], antimicrobial, anti-inflammatory [6], antioxidant, and significant antitumor activity [7], memory enhancement effect [8], and anxiolytic activities [9].

Moreover, skin whitening [10], skin depigmenting [11], antiaging and antierythemic [12] activities, an emollient [13], an antiacne [14] activity, a potential cancer chemopreventive agent [15], cardioprotective effects [16], restoring liver function in patients suffering from hepatitis C [17], antidepression [18], hepato protective [19], and atherosclerosis [20] and photoprotection effects [21] could be mainly provided by using licorice extract.

The medicinal and pharmacological uses of licorice ingredients have been described in several studies [22–24]. Licorice contains a variety of substances such as sugars up to 18%, flavonoids, saponoids, amino acids, sterols, gums, and starch. The effective ingredient in licorice is mainly glycyrrhizin, which has antiviral, anti-inflammatory [6] and antioxidant properties [25] and a triterpenoid glycoside, which constitutes up to 14% of total soluble solids content [26] giving the characteristic sweet taste from the licorice root. Glycyrrhizin has few calories and can be used in the form of ammonium glycyrrhizin or monoammonium glycyrrhizin in nutrients. There are a lot of efficacious compounds in licorice roots like licochalcone known as a novel estrogenic flavonoid isolated from herb licorice root that was reported to show significant antitumor activity in various malignant human cell lines [7].

The licorice root extract has been widely used in the nutriment industry as a sweetening agent as ammonium glycyrrhizin is about 50 times as sweet as cane sugar [27]. There is a growing commercial interest in using licorice root extract in food foams. Foaming properties of licorice extract influence the sensory quality and shelf-life of the final product [28]. So, the licorice has found widespread usage as a foaming agent in beverages [29], in halva and sweets [30, 31]. Commercially, the licorice root extract is supplied in concentrated or powdered form for the ease of transportation [26].

Biologically active separation of natural products from useful plants or medicinal herbs is of great interest to the pharmaceutical and food industries. The separation techniques known as solvent extraction or supercritical fluid extraction are usually employed for isolation of valuable ingredients. Because of the ecosystem harm of organic solvents used, an alternative method of general interest is foam fractionation called a method based on adsorptive bubble separation technique. For the enrichment of surface active substances, gases (e.g., nitrogen, oxygen, air, and carbon dioxide) are introduced. So, enrichment was formed into the foam [32]. The separation technique for biological effective compounds of foam fractionation by adsorptive bubble chromatograph is more effective, especially at low initial concentrations (up to 1 × 10^10^ mol/L) of substances [32–36]. It is suitable method for the quantitative and effective enrichments in comparison with other traditional methods mentioned above [37]. No study has been done about enrichment of chemical composition from* Glycyrrhiza glabra* roots with bubble separation techniques so far. So, the objective of this study was to enrich the biological effective fractions by adsorptive bubble separation technique.

## 2. Experimental

### 2.1. Solution and Reagents

The chemical solvents used were of spectroquality grade for HPLC and analytical grade. All of the chemical solvents were obtained from Sigma Chemicals Co. such as glycyrrhizic acid ammonium salt as HPLC standard and *β*-lactoglobulin, albumin bovine, and starch (soluble) as foaming agents. The water used in experiments was purified using Milli-Q System (Millipore Corp.).* Glycyrrhiza glabra* L. roots were purchased from a local market.

### 2.2. Preparation and Extraction of Licorice

The licorice powder (10 g) was mixed with 300 mL distilled water. Mixtures were heated to 60°C under stirring for 4 h and, after cooling down, the solution was filtered by using a fluted filter. Finally, the licorice extract was stored in the refrigerator at 4°C until the absorptive bubble separation.

### 2.3. Adsorptive Bubble Chromatograph with Different Additional Substance

**Figure 1:**
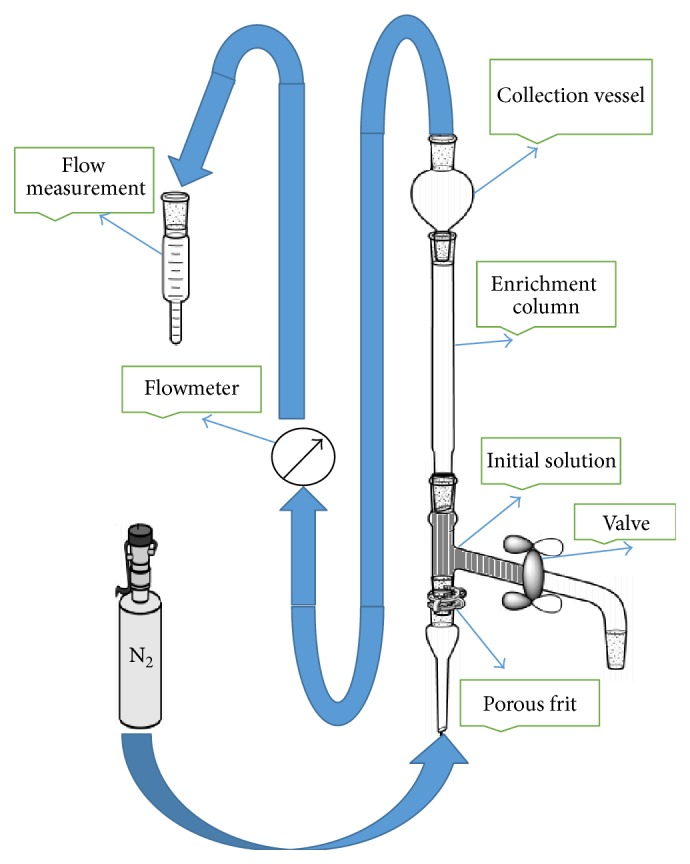
Scheme of the Adsorptive Bubble Separation Apparatus.

As shown in Figure 1, the equipment was established with a glass column (ID 18.5 mm, length 15 cm) with a porous frit (P 3, porosity 16–40 *μ*m), flask (250 mL), initial solution column (ID 18.5 mm, length 15 cm), and volumetric flask (for flow measurement mL/min). The attention was taken that the column was highly clean and that the ground glass was free of fat. Gas bubbles were created by passing a stream of nitrogen through a glass frit dipping into the liquid pool at the bottom of the column. The bubble rises up the column and the liquid part of the bubble drains due to gravitation back to the initial solution. In order to increase the foaming ratio, the foaming agents such as *β*-lactoglobulin, albumin bovine, and starch (soluble) were used in this study. The foam was collected in collection vessel at the upper part of apparatus. After bubble separation, enrichment samples collected in collection vessel were weighed. The separation takes place by means of an optional adsorption at the liquid gas interface of raised bubbles.

In the experiments, four bubble separation parameters were used with *β*-lactoglobulin, albumin bovine, and starch (soluble) preferred as foaming agents and without additive. For each experiment, 70 mL of licorice root extraction amount was prepared, containing 30 mg foaming agents in 120 min foaming time. The carrier gas was nitrogen with a flow rate of 30 mL/min. The pH value was also 2.5. The enrichment ratios (*E*) were calculated as follows:

(1) $$\begin{array}{c} E=\frac{C_{foam}}{C_{Start}}, \end{array}$$

where *E* is enrichment ratio, *C*_foam_ is concentration (*μ*g/mL) of foaming after enrichment with bubble separation, and *C*_start_ is initial concentration as *μ*g/mL. The eluting fractions were collected at fixed intervals and after foam destruction with 1 mL eluent (*A*), used in HPLC as mobile phase subjected directly to analysis.

**Figure 2:**
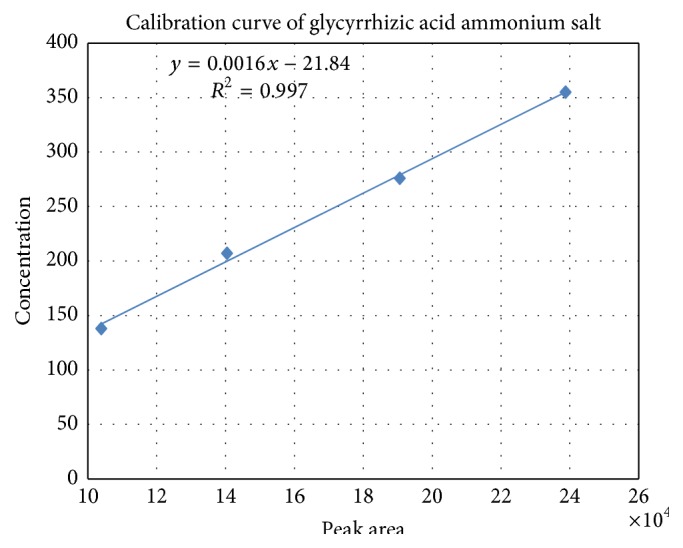
Standard curve of reference compounds on HPLC.

All the calibration curves were plotted based on linear regression analysis of the integrated peak areas (*x*) versus concentrations (*y*, mg/L, ppm) of the reference solution at four different concentrations. Regression equation, retention time, correlation coefficient, and standard curve of glycyrrhizic acid ammonium salt in HPLC were shown in Table 1 and Figure 2.

The starting, remaining solution and extract foamed samples were analyzed by HPLC for identification and quantification of glycyrrhizic acid ammonium salt.

As shown in Table 2, the correlation coefficients for all extracts were plotted based on linear regression analysis of the integrated absorbance value (*x*) versus concentrations (*y*, mg/mL) of the reference solution at four different concentrations. As seen in correlation coefficients, the calibration curves plotted in the different concentration were on the linear for all the experiment and the results showed that the extract has small standard deviation.

### 2.4. HPLC Study

The HPLC analysis was performed on Gynkotek 480 equipped with Rheodyne 8125 injector and 20 *μ*L sample loop, a Gynkotek UV-detector (UVD 340 s; wavelength was selected as 254 nm), Kromasil 100 C18 columns (Knauer, Germany: 5 *μ*m, 25064.6 mm, column temperature 258°C), and Uniflow Degasser DG-1310. The eluent contained 210 mL of methanol, 210 mL of acetonitrile, 174 mL of distillated water, and 6 mL glacial acetic acid without gradient elution, at a flow rate of 1 mL min^−1^.

## 3. Results and Discussion

The licorice root extracts gave plentiful foam during bubble separation, when *β*-lactoglobulin, albumin bovine, and starch (soluble) (30 mg for each experiment) were used as foaming addition in licorice extract. The foam without addition of a surface active substance was weak in the licorice extract. This situation was probably due primarily to the reduction of surface tension.

The licorice root extraction amount, pH value, foaming agent amount, foaming time, and gas flow rate were taken as optimum on the enrichment. The foaming agent type was used as variable, while other parameters were stable. The results given in Table 3 illustrate that the higher or lower enrichment rates of glycyrrhizic acid ammonium salt into the foam fraction from licorice extract can be observed during all experiments. Varying the additional substances influenced the enrichment ratios and yields.

As clearly shown in Table 3, the foam separated licorice extract treated with *β*-lactoglobulin has the highest enrichment ratio of glycyrrhizic acid ammonium salt. Moreover, the enrichment ratio of the glycyrrhizic acid ammonium salt from licorice extract treated with *β*-lactoglobulin is as much as 368,3 times higher compared to that of initial concentration. The lowest enrichment ratios were determined from the separation without additive as much as 5,9 times lower than that of initial concentration. Besides, no significant difference in the enrichment ratio is determined between bubbling processes with soluble starch and without any foaming agent. About 62 and 16 times' increments are recorded due to addition of *β*-lactoglobulin and albumin bovine used for foaming, respectively. The *β*-lactoglobulin method finds highest enrichment ratio for glycyrrhizic acid ammonium salt and may be preferred as enrichment method in the bubble separation techniques in industry because of the highest yield.

## 4. Conclusions

The glycyrrhizic acid ammonium salt was successfully enriched by* Glycyrrhiza glabra *L. root extract quantitatively by isoelectric focused adsorptive bubble separation technique with and without foaming agents. The findings showed that *β*-lactoglobulin used as a foaming agent has the highest enrichment ratio of glycyrrhizic acid ammonium salt compared to initial concentration and other foaming agents on the adsorptive bubble separation technique. The bubbling process without any foaming agent was substantially observed to have the lowest enrichment ratio compared to the ones with foaming agents such as beta-lactoglobulin, albumin bovine, and starch (soluble). Besides, the bubble separation enrichments with and without starch used for foaming did not give any significant difference. Therefore, transfer of effective compound to the foam from solution in the presence of *β*-lactoglobulin and albumin bovine was more efficient compared to that of aqueous licorice extract.

## Figures and Tables

**Table 1:** Regression equation, retention time, and correlation coefficient of reference compounds on HPLC.

Number	Sample concentrations (mg/L) (ppm)	Retention time (min)	Regression equation^a^	Correlation coefficient (*r* ^2^)
(1) (2) (3) (4)	138 207 276 355	3.04	*y* = 0.0016*x* − 21.842	0.9917

^a^ *x*: peak area of components, *y*: concentration of components.

**Table 2:** Correlation coefficient, enrichment addition, type, and volume of all the sample types on HPLC.

Number	Enrichment addition	Sample type	Sample volume (*μ*L)	Distilled water volume (*μ*L)	Correlation coefficient (*r* ^2^)
(1)	*β*-Lactoglobulin	Start extract	25, 50, and 100	975, 950, and 900	0.9941
(2)		Extract foamed	300, 500, and 1000	700, 500, and 0	0.9978

(1)	Albumin bovine	Start extract	25, 50, and 100	975, 950, and 900	0.9943
(2)		Extract foamed	300, 500, and 1000	700, 500, and 0	0.9996

(1)	Starch (soluble)	Start extract	25, 50, and 100	975, 950, and 900	0.9949
(2)		Extract foamed	300, 500, and 1000	700, 500, and 0	0.9978

(1)	Without additive	Start extract	25, 50, and 100	975, 950, and 900	0.9951
(2)		Extract foamed	300, 500, and 1000	700, 500, and 0	0.9998

**Table 3:** Enrichment ratios and start, foam, and residual extract concentrations (mg/mL) of *Glycyrrhiza glabra* with *β*-lactoglobulin, albumin bovine, and starch (soluble) preferred as foaming agents and without additive.

Experiment number	Foaming agent method (mg)	Initial concentration (*μ*g/mL)	Foam concentration (*μ*g/mL)	Enrichment ratio (ER)
1	*β*-Lactoglobulin	12 ± 0.986	4420 ± 183.43	368.3
2	*Albumin bovine*	24 ± 1.982	2170 ± 90.055	90.4
3	Starch (soluble)	348 ± 29.884	3260 ± 135.29	9.4
4	Without additive	360 ± 33.88	2100 ± 87.15	5.9
